# Stewardship in Health, Designing a Multi-Layer Meta Model: A Review Article

**Published:** 2019-04

**Authors:** Taha NASIRI, Amirhossein TAKIAN, Shahram YAZDANI

**Affiliations:** 1. Department of Health Management and Economics, School of Public Health, Tehran University of Medical Sciences, Tehran, Iran; 2. Department of Global Health and Public Policy, School of Public Health, Tehran University of Medical Sciences, Tehran, Iran; 3. Department of Medical Education, School of Medical Education, Shahid Beheshti University of Medical Sciences, Tehran, Iran

**Keywords:** Stewardship, Governance, Leadership, Model

## Abstract

**Background:**

In the current study, we aimed to explain the concept of stewardship by employing a critical review perspective and, finally, to develop a model.

**Methods:**

Critical review method and Carnwell and Daly approach and using particular keywords related to stewardship, and searching databases were used. In the initial search, until 2018, 1050 studies were obtained, which using targeted sampling method, 32 studies were finally selected. Then, selected studies were criticized and finally, and the conceptual model of stewardship was designed and explained.

**Results:**

After reviewing and criticizing; the concept and aspects of the model were categorized in the sub-tasks of knowledge-generation, strategic framework, evidence-based policy-making, system design, resource allocation, capacity building, enforcement/alignment, that through operation leads to achieving goals. Final model was categorized into three effective levels.

**Conclusion:**

Some studies mentioned the governance and stewardship as same concepts, while our results showed that policy-making, governance and leadership, and many other concepts, can be categorized as a part of stewardship.

## Introduction

Governments are responsible for the well-being of their citizens. Stewardship is the heart of policy-making for health systems. Stewardship affects policy making at different levels, particularly at higher levels (1). Stewardship is mentioned in religious books, in Genesis God has created humans as the steward of the Universe. The Old Testament introduced the Joseph as the steward of the Egyptians means a person who has a high level in democracy but acts as a servant of his nation (2). WHO report has defined the stewardship as one the function of governments that are responsible for the well-being and welfare of their societies. The reported also referred to the trust ability and legitimacy that citizens attach to the government’s activities (3–5).

In addition to what mentioned about different definitions and aspects of stewardship, which reveal the importance of stewardship in health system, developing countries do not have a desirable status in health system stewardship, that seems to be one of the main reasons for poor knowledge and weak awareness of researchers, policy-makers and managers about stewardship concept –and its aspects- and finally lack of an operational model to implement stewardship in health system (5).

The government responsibility was defined as a mean to increase society’s welfare as its steward (2). On the other hand, WHO 2000 report defined the stewardship as exact and responsible management of people’s welfare and mentioned three main tasks (1). Stewardship is the responsibility of the government so that usually ministries of health in collaboration with other organizations and institute do it (6). Pan America Health Organization mentioned eleven tasks as the main tasks of stewardship (7). Ministry of health is the main steward of people’s better living that needs effective decision-making (8). While emphasizing on the ambiguity of governance concept, noted that stewardship and governance as equal concepts and acknowledged that they are sometimes used interchangeably (4, 9–11). Some authors have also introduced leadership issues to this area (12). Despite these definitions, there is a conceptual ambiguity in the concept of stewardship. This issue and other mentioned problems inspired us to identify the nature, dimensions, and tasks of health systems stewardship.

Therefore, the current study aimed to criticize other studies related to stewardship concept, in order to clarify its trend during the past two decades and providing a model to help policy-makers in terms of appropriate stewardship of health system. The current study by using critical review, which includes identifying different aspects of the stewardship models, criticizing and reviewing it by authors of paper and other studies, tried to explain the stewardship concept and finally, to provide a model.

## Methods

Since the main intention of the authors was to have some conceptual innovation and synthesis, critical review method was employed.

Critical review aims to show the wideness of research on a specific issue and to critically assess it. It is more than simple description of identified studies and includes some degrees of conceptual analysis and innovation to identify important issues, and typically results in developing a new model. The resulted model may be a synthesis of current models or a new interpretation of available data. Mentioning methods of search, synthesis, and analysis is not necessary (13).

This research reviewed literature through using Carnwell and Daly approach (14) and specialized keywords related to stewardship in the PubMed, Google Scholar, Embase, Elsevier, Emerald, Scopus, Iran Medex and SID databases as well as Google until 2017.

Initially, a comprehensive and high sensitive search was conducted via Google, then each database was searched using its own search methods. To increase validity of samples, a series of stewardship related keywords, including stewardship, governance, policy-making, and leadership were used. In addition, reference list of papers and books were searched by using these keywords. In order to expand the search process another set of keywords, including health system, health policy, health sector, health care system, were added to the search strategy.

Based on the topic and abstract, initial screening was performed by one of the reviewers (TN). Second reviewer (SY) independently reviewed selected papers, and in a case, which there was disagreement, consensus was achieved. At first phase, about 1050 papers were examined, which 110 of them were chosen and reviewed selectively at the second phase.

Literature that had more rich literature (if they mentioned to the definition and interpretations of the stewardship and other related concepts, concepts that are close to it or contrast concepts, and similar constructs), new references, prestigious of journal or database, citing to valid references were used and references which investigated the concept of stewardship in other fields were removed.

Therefore, studies were ranked and were examined respectively. The investigation and criticization of studies using a qualitative research approach were continued to the saturation point (13, 14). Finally, in the third phase, 32 studies were critically evaluated. Studies were reviewed by two independent reviewers, they were presented in a group that authors of the current study (SY, AT, TN) were a part of that. By employing critical review, main concepts were extracted and then were presented to a group comprised of the authors of the current study. While explaining the stewardship concept, its aspects, infrastructure and tasks in the health system are explained, and finally, the conceptual model of stewardship in the health system should be designed and explained.

## Results

### Conceptual model of Saltman and Ferroussier-Davis

Stewardship concept implicitly refers to the government responsibility to promote population welfare, focus on trust and legitimation of activities seen by individuals (2). In contrast, Kass noted the trust of people in business affairs as the key for serving (15). Armstrong mentioned to the “self-actualizing civil servant”, as an individual who service people with all of abilities and potential talents, not a person who is trying to satisfy personal desire for power and positions. Saltman and Armstrong's views emphasize values, ethics, legitimation, presence of law and trust to improve the society’s welfare. Whereas, Kass did not emphasize these concepts (2). Unlike, economic view of the Agency theory believes in economical human, and consider subordinates as an opportunity to make more profit. While, the stewardship concept, like participatory, interactionalist, servant and value-based approach to power, consider subordinates as pluralistic, organizational and trustworthy (16). On the other hand, stewardship is a model of governance, that seems not to be true and is different from a view that considers it as a task of health system. The point is that Armestrong and Davis defined the steward (as a part of society), instead of stewardship. Indeed, their focus is on human, while new views and studies focus on systemic view.

### The world model of 2000

WHO’s definition, as one of the first ones, defined the stewardship as one of the government functions that have the responsibility of society’s well-being and welfare, and cares about the level of trust and legitimacy that citizens have for government activities. Stewardship tasks are: forming health policies, developing a vision and strategies, influencing through the control and regulation approaches, using collective intelligence, so effective function of these three components is a guarantee for effective stewardship. The first two emphasize on the responsibility, in order to monitor the decision-making process and other health stakeholders, advocacy, guiding, consultation, coordination. The latter emphasizes using information and evidence in decision making. While, Saltman view did not consider involvement of other stakeholders in the health sector and, also, did not consider a role for leadership of national government, as in the WHO report. In contrast with Slatman’s view that is a ‘servant’ approach, WHO 2000 report did not mention of it and Ministries of Health (MoHs) have the main responsibility in stewardship (2, 3). Again, in contrast with Slatman’, the WHO report did not mention the level of laws, trust, legitimacy, value, and ethics as necessary factors for governments. Categorizing stewardship task in the WHO report is not sufficient and needs to be expanded to cover all aspects and dimension of it.

### Travis et al. model

Stewardship is one of the government’s responsibilities, usually done through MoHS. Responsibilities of different aspects of stewardship can be divided between national and local sectors, ministries, commissions, specialized associations, inspectors, insurance funds, purchaser's agents and other providers. But, government through the MoH still acts as the steward of stewards and guarantee the comprehensive implementation of stewardship. Stewardship contents assurance from monitoring, regulation, and being accountability of all stakeholders of health system in four main tasks of health systems. Governance principles are developed by the government and are employed to improve government’s performance. Travis differentiated between stewardship and governance so that the quality of governance influences the environment which health measures are performing. Steward of the health system has the responsibility to monitor the performance of health with regard to the governance principles. Often six sub-function are listed for stewardship (6).

Saltam defined the stewardship as a function of governance, that government has the responsibility of stewardship in health (2), while, the WHO report emphasized on the role of MoHs as the stewards of health systems (3), that is different from Travis view on stewardship (6). Commonwealth countries considered transparent, being responsible and participation as the main concepts of good governance, that in terms of number and content are different from what is mentioned by Travis and his colleagues. Indeed, according to this definition stewardship is a sub-set of good governance (17). Moran noted three main concepts of creating valid decisions, creating a mean to influence decisions, and advocacy as health governance. This view contains a small part of the main tasks of stewardship, including advocacy, policy-making, and strategic orientation (18) that in terms of using equivalent words, is different from Travis view. Travis did not mention the concept of policymaking. Although stewardship is the main responsibility of government and all public and private organizations involved in health or more generally all administrative structure of a country, Moh is the steward of stewards. This view is different from the view of Moran and commonwealth countries.

### PAHO regional office model

This model comprises of 11 Essential Public Health Functions (EPHF) (7). EPHF have some measurement tools. Travis believes that stewardship arena is wider than what is mentioned in EPHF and do not contain some functions of EPHF such as provision or resource generation (like human resource training) (6). This model did not introduce specific categories of stewardship, like those in Travis or WHO report and, also, did not consider the roles for ministries and/or other organizations and interactions.

### Boffin’s model

Boffin has noted six dimensions for stewardship. Depending on the used definition, governance in most of the times is equivalent to stewardship. Steward’s activities to promote health are not against governance principle (4) that is different from Travis view, which MoH is the steward of stewards (6). Murray and Frenk noted three main aspects of stewardship: 1) arrangement, implementation and monitoring of health laws; 2) ensuring that a level is playing its part among all players (provider, patient, and purchaser); and 3) strategic orientation for health system (18). These tasks are narrower than those in Boffin and Travis views. Stewardship concept is almost equal to governance, and just that stewardship can have a better reaction to elements of system orientation and is considered as a wise task, while governance emphasizes on structural aspects of activities and procedural concept, that is different from Boffin view. Boffin’s view about the equivalence of stewardship and governance is different from other researchers. On one hand, he has added the function of performance assessment to the list of governance tasks. Indeed, the stew-ardship approach is macro level, human-center and contains accountability, strategic orientation and finally people health, but governance includes managerial procedures and systematic aspects (16). This means that stewardship and governance are two different concepts (i.e. governance is a sub-set of stewardship).

### Alvarez-Roseto and colleague's model

Alvarez-Rousto et al believed evidence-based policy-making as stewardship task and this role are seen as steward of evidence from the MoHs. In contrast with Travis view, often, Alvarez-Rousto et al interchangeably used stewardship and governance concepts. Health system governance first refers to the complex arrangements of health system management. Stewardship implicitly refers to wide accountability of the health system and, finally, to the health of people. Therefore, MoH’s cannot be the stewards of other sectors (9). Thus, Travis’s view, steward of stewards, is different from Alvarez-Roseto, evidence-based stewardship. Smith et al, in contrast with other approaches, added the leadership and governance words to the concept of stewardship to find an operational concept for it, which is different from Alvarez-Roseto’s view (12). Governments not only are the stewards of health systems but stewards of health enhancement factors such as education, employment, transportation policies and so on, which affects health through economic and social factors (9). This view is different from Boffin and Alvarez-Roseto, equivalence of stewardship and governance. Alvarez-Roseto had a different and limited view to stewardship, and finally, they have mentioned the accountability as the most important concept of stewardship, which based on Travis and Boffin views, it is only a part of stewardship. By Alvarez-Roseto view, only the MoHs are stewards of evidence to policy-making, which is an irrational and limiting view toward the difficult task of stewardship.

### Siddiqi’s model

In most of the times, stewardship and governance are used interchangeably. And, in contrast with WHO 2000 report, he considers the governance as one of the functions of the health system. This view is different from Travis and Veillard, which consider the MoHS as the steward of stewards. Siddiqi’s model, that is a framework to assess the governance in health system, comprises of 10 governance principles at three levels, national, policy-making formulation, and policy implementation. Regardless of the concept of stewardship term, he mentioned to more dimensions of the stewardship concept, such as efficacy, effectiveness, and equity, while other models such as WHO ignored these principles (10).

Siddiqi assessment categorization is different from the World Bank categorization and contains issues such as violence control and political stability. They had different views, so that World Bank view is based on the frame of voice and accountability and people’s presence and their voice through civil society organizations, and the association that Siddiqi assessment categorization has with World Bank framework to evaluate governance and its link with outcome development; these are some of the prominent difference between these two frameworks (19). United Nations Development Program has considered the five principles of good governance (10) that is different from Siddiqi’s view in terms of categorization. The need to consider ethical aspects and research are from necessities of health system governance, including independence, nonmaleficence, benefit and equity, not included in the UNDP view. Based on what’s mentioned above, Siddiqi’s view provides a framework to evaluate health system governance, and in this view, it can be considered as an evaluation model–he provides a different viewpoint than existing studies which he considers stewardship as a subset of good governance. Based on this view, unlike others, MoH is only responsible to ensure that the health system goals are met, which seems irrational because intra-sectoral collaboration with other ministries is needed.

### Veillard’s conceptual model

The government, particularly MoH, is responsible for health system outcomes and better living of the population to protect their interests, which needs effective decision-making based on accountability, transparency, and systematic design. One of the criteria of people trust is considering ethical aspects and values. He has introduced six aspects of stewardship tasks, which are consistent with those introduced by European member states of WHO, and leads to achieve the final goals of the health system.

The borders of stewardship in the health sector cover intra-sectoral factors, such as socioeconomic determinants of health (20). However, Veillard’s operational framework introduced the MoH as the steward (8). Davis noted that health system stewardship contains health system related strategies (strategic management) and policies. He described health system stewardship boundaries of MoHs in three levels of strategies, policies and main tasks of health systems, factors which influence health, such as education, employment, trading, and tertiary factors, such wider socioeconomic factors (the latter affects the other two) (21). This view reveals a macro approach to stewardship considered increasing and socioeconomic factors as a tasks of stewardship beyond the authority of MoH, as the main steward, which needs to collaborate with other organizations. This view is not consistent with Veillard’s model that restricted stewardship to the MoHs. Indeed, Veilard’s model did not consider operational problems and operational view in countries with different values and governance backgrounds has some limitations. While a few countries are able to implement all aspects of health system stewardship.

### Erica Barbazza and Juan E. Tello conceptual model

Governance concept is ambiguous so that there is no consensus on that, but social and economic contexts and players role, both internal and external, contributed to its evolution. However, there is a general consensus that governance tasks are a set of procedures (customs, law, and policies) which formally and informally influence the distribution of responsibilities and accountabilities of health system players. Governance and leadership are Subdivisions of stewardship and include the existence of a strategic policies framework in combination with effective monitoring, creating coalitions, regulation, attention to system design, and accountability. There are major challenges in globally accepted definition of stewardship, between stewardship concept, governance and leadership. This task of system stewardship contains a definition that includes developing a vision for health system and borders which players act on them. The aspects that he has introduced in most of the times are the same for stewardship but only are mentioned in another way. While Veillard has a broad view in the arena of values and contexts, seen all of his models, Barbazza noted that these values comprise of corruption control, democracy, human right, ethics, and honesty, preventing conflicts, public good and the rule of law. In contrast with Travis view, Barbazza believed that stewardship and governance are similar concepts (11). Kickbusch emphasized the existence of governance and leadership in the framework of health system strengthening. The Ministerial Conference in Estonia and recently the European region emphasized on its issue as 2020 Health. Noting these concepts show that governance and leadership are elements of stewardship, which is different from Barbazza’s view (22).

Barbazza did not provide an exact definition of governance applications and only mentioned a series of tools for strengthening governance tasks for each dimensions. Moreover, while mentioning system’s boundaries, he has determined instruments that transform health sector (that its primary goals is improving health) to health system (one of its goals is improving health), which stewardship determines them. The association between values and tasks is not clear, and in some instances, value and governance task are the same and there is no explicit distinction of concepts. There are no defined roles for MoHs and other ministries to do health tasks. There is a difference between governance, leadership and stewardship concepts, that is in opposite of Barbazza (Table 1).

**Table 1:** Aspects of the models

***Sub-functions* Barbazza**	***Veillard***	***UNDP***	***PAHO (EPHF)***	***World Bank***	***Boffin***	***Siddiqi***	***Travis***
Accountabilities	Strategy formulation and policy development	Legitimacy and voice	Monitoring, evaluation, and analysis of health status	Voice, accountability, political instability, violence	Overall system design	Regulation	Generation of intelligence
Partnership	Intersect oral collaboration and action	Direction	Quality assurance in personal and population-based health services	Government effectiveness, Regulatory burden	Performance assessment	Consumer Support	Formulating strategic policy direction
Formulating policy/strategic direction	Health system governance and accountability	Performance	Health promotion	The rule of law, corruption control	Priority setting	Strategic vision	Ensuring a fit between policy objectives and organizational structure and culture
Generating information/intelligence	Attention to system design	Accountability	Social participation in health		Intrasectoral advocacy	Participation and consensus orientation	Ensuring a fit between policy objectives and organizational structure and culture
Organizational adequacy/system design	Health system regulation	Fairness	Development of policies and institutional capacity for public health planning and management		Regulation	The role of law	Building coalitions / Building partnerships
Participation and consensus	Intelligence (data and analysis) generation		Strengthening of public health regulation and enforcement capacity		Consumer Support	Transparency	Ensuring accountability
Regulations			Evaluation and promotion of equitable access to necessary health services			Responsiveness	
			Human resources development and training in public health			Equity and inclusiveness	
			Research in public health			Effectiveness and efficiency	
			Reduction of the impact of emergencies and disasters on health			Accountability	
						Intelligence and information Ethics	

### Conceptual model of stewardship in health

The authors of the current study concluded that three categories of factors can influence health: 1) social determinants of health; 2) risk factors for health, and finally, 3) the health system itself. Although this model comprises of three levels of wider economy and society, health-related external sectors, and health system affecting by three above mentioned categories. This study tried to provide a general model of stewardship in health system, and a conceptual model in three levels of wider economy and society, health-related external sectors, and health system.

### General model of stewardship

Intelligence generation, strategic framework, system design, resource allocation/development, and capacity building, evidence-based policy-making, alignment of policy with operation (Enforcement/Alignment) are considered as sub-functions of stewardship included in the general model:

### 1. Intelligence generation:

It emphasizes on the necessity of using information and resource information in decision-making for all stakeholders of the health system (6) and comprises of stewardship, knowledge generaon, knowledge management, knowledge dissemination, environmental scanning, problem identification, and needs assessment in order to respond to the needs (23).

### 2. Strategic framework:

Means developing a criterion for decision-making and strategic direction in policy-making context and includes setting reference values, determining criteria system, setting strategic directions, and strategy formulation (24).

### 3. Evidence-based policies/decisions :

Precise policy-making based on rational conceptual information generally accepted, because evidence-based policies which come from the society are the prerequisite of stewardship concept realizations (25).

### 4. System design:

It means establishing infrastructures that provide better situations for government or health system’s performance (11).

### 5. Capacity building:

Creating situations that by doing these tasks, whether in management or training aware human resources areas, the context for better performance of stewardship tasks became available (26).

### 6. Resource Allocation / Development:

Consider the health budgeting and determining a share of the budget for health (27).

### 7. Enforcement/alignment of policies with operations:

The Health system as the steward of health is responsible for different policies, including governmental, operational and clinical policies expected to convert to an operation, which finally improves the health (27).

A major problem in many health systems is the alignment of operations and policies that stem from a defect in governance. To increase alignment between operation and sectoral policies of the health system, concepts such as defining standards, monitoring and Regulatory activities (rewarding and punishing) must be used. Organizational accreditation, work permit, and financial flow are examples of regulatory tools. Indeed, all mechanisms employed to align Intra-Sectoral Operation (ISO) with health Intra-sector policies are called governance. The Health system steward as a part of government can monitor and review actions of ISO, but its inter-sectoral activities are limited. Therefore, leadership mechanisms must be employed. Leadership is defined as using legitimized instruments that influence on other sectors to achieve aims and goals of the health system. Therefore, it contains stakeholder analysis, advocacy, conflict negotiation, partnership building. Then, alignment of policies and operations are revealed in the convergence governance and leadership concepts (27, 28).

**Fig. 1:**
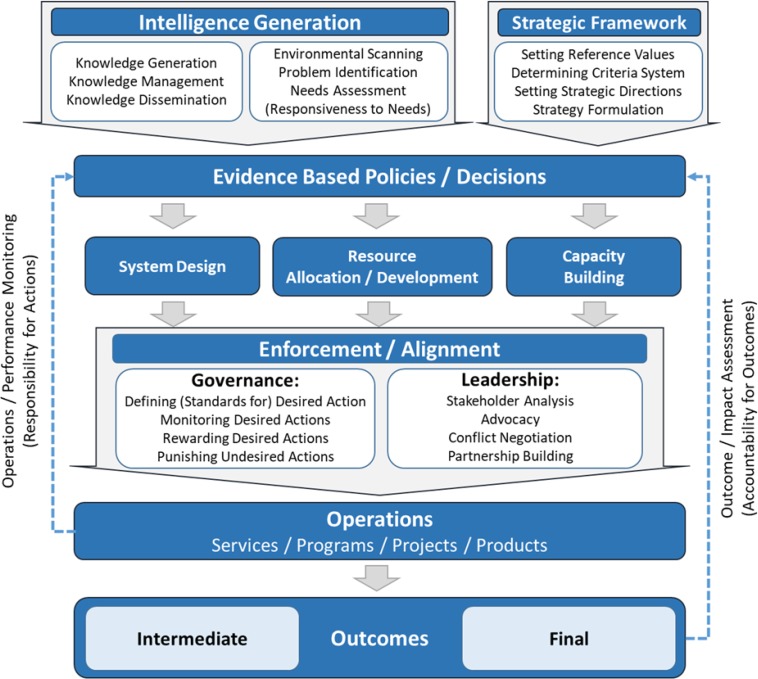
General model of stewardship

These stewardship sub-functions turn into health goals in middle and final levels through health-related operations. Evaluation of outcomes and achieving goals leads to accountability of outcomes and, monitoring the actions results in responsibility for actions. Stewardship is performing, in three levels of wider socio-economic level, health-related extra sector and health system to achieve final health goals. All of these levels need transparency, policy stability, quality of bureaucracy (i.e. appropriateness of bureaucracy), and rule of law to have better performance, and will have the desired performance if these issues form a platform. All of these tasks turn into middle and final goals of health in a process (4, 6, 10, 11, 19, 29) (Fig. 1). These sub-functions have different subset at each of the three levels.

### 1. Wider economy and society level

**Fig. 2:**
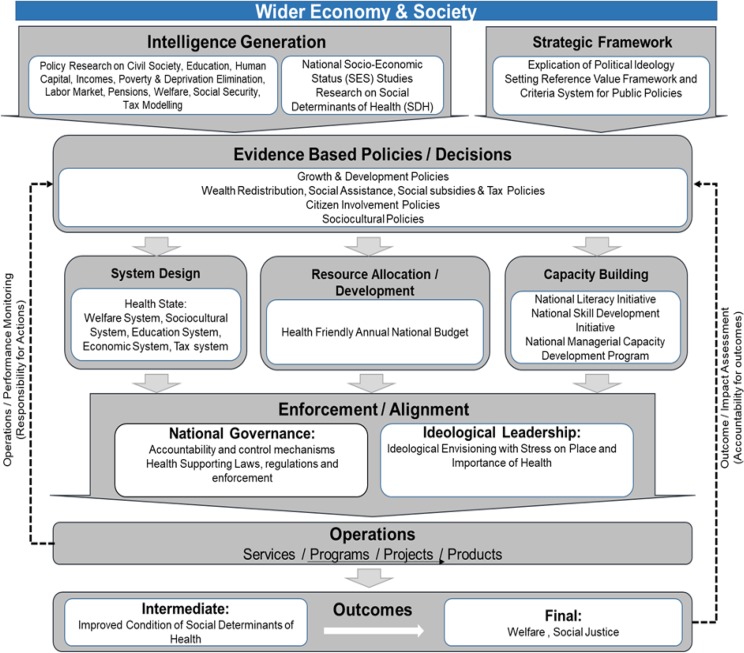
Wider economy and society level

Attention to Health Risk Factors (HRFs) is one of the achievements of health systems. HRFs have common cause roots, including poverty, deprivation, illiteracy, discrimination, and injustice, are called Social Determinants of Health (SDH). Social determinants, on the one hand, influence HRFs and on the other hand directly influence health and diseases. Attention to SDH, even one step before HRFs, can ensure maintenance and equitable promotion of health. The other point is that improving SDH simultaneously improve physical, mental and social health. Therefore, regardless of the country, social and economic factors have a major effect on health. At the macro level, all parts of government should work in line of health (26, 27, 30–33) (Fig. 2).

### 2. Health-related extra sector

**Fig. 3:**
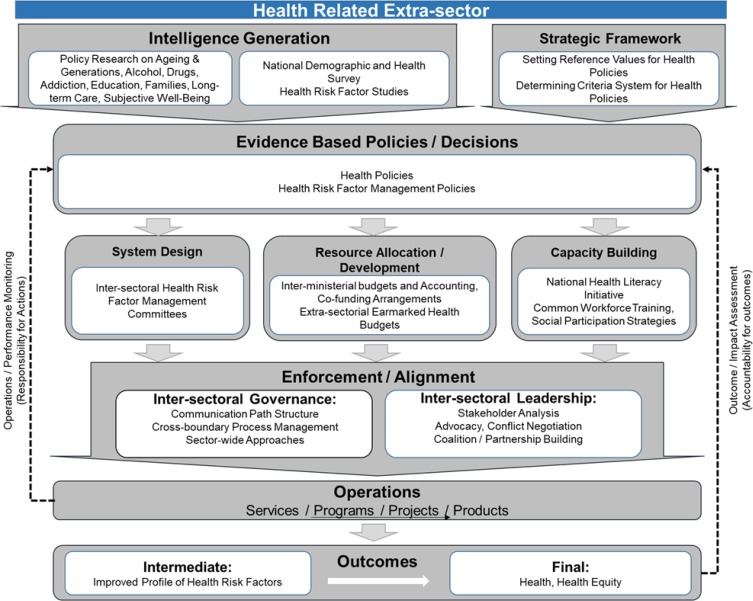
Health-related extra sector level

Countries need systems which protect their citizens against health risks. HRFs are categorized into three: 1) genetic; 2) environmental risk factors, and 3) behavioral risk factors. Environmental and behavioral particularly have direct impact on diseases occurrence. Usually, a significant burden of diseases in each county can be attributed into few HRFs. A significant part of these HRFs are related to the factors that are not in the control of health systems. All ministries, organizations, institutions, and sectors that their secondary goal is health and improving it, such as agricultural sector and ministries related to water issues, are health-related extra sector. Therefore, HRFs management is important at this level. The main difference between this part and previous one is that in this level criteria back to the health policies, while in the previous part, must be determined at level of public policies (27, 26, 30–34) (Fig. 3).

### 3. Health system

**Fig. 4:**
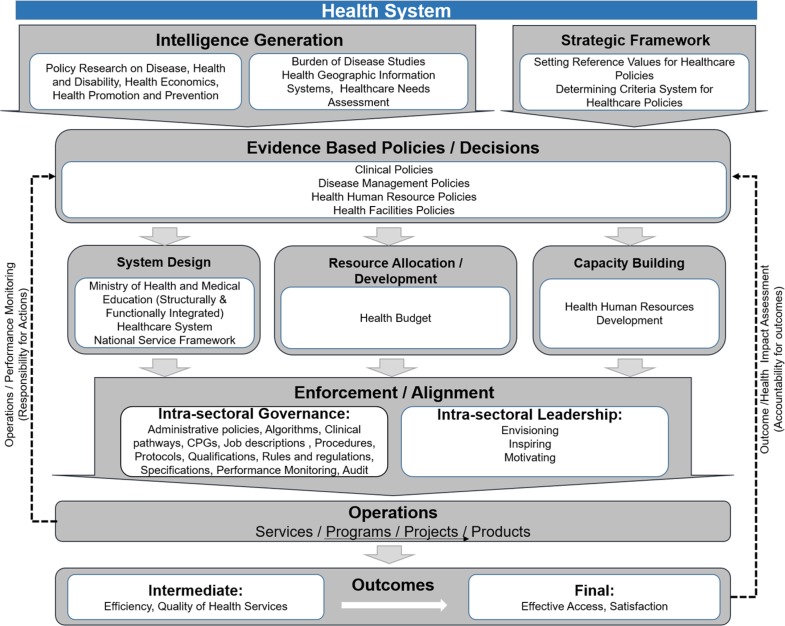
Health system

Almost all countries around the world have a health system, but existence or non-existence and/or its extent depends on the level of coordination and goals of different parts of the health sector. In the first look, it may seem that the health system only comprises of public sector. But this is not true, because in many undeveloped or developing countries the public part of health sector even is not coordinated or targeted so that they cannot be called health system. In this part, the main focus is on healthcare provision and refers to all parts which one of their goals is improving health (26, 27, 30–35) (Fig. 4).

## Discussion

In this study, we tried to critique the nine major studies related to the subject and many related studies to clarify the concept of the stewardship and finally provide a related model. The point is that, although stewardship elements are described in studies such as Boffin and Siddiqi, Alvarez and Barbazza, but they believed that stewardship and governance are equal concepts, but an in-depth investigation indicated that they actually have used the stewardship concept, as authors of the current study emphasize on that (4, 10).

All of these studies except Travis did not investigate the levels which influence health. Davis in his study described these levels as task borders (23). Three influential levels, wider economy and society, health-related extra sector and health system, described by the model, can be regarded as a guide to operations stewardship in health. A policy-making concept can be defined for evidence-based policy-making (i.e. determining strategy and macro direction of health sector) if be used along with intelligence generation and explaining a strategic framework, that is so important. Except for Roseto and et al. which mentioned this issue as evidence-informed health policy, none of investigated studies investigated this issue (9). Emphasize on policy-making, leadership, and governance as the main elements and as a sub-set of stewardship, are among distinguishing points of this study that comprises elements. In implementation area, we are faced with capacity building, resource allocation, and system design, which investigated studies only mentioned the latter (4). Alignment/enforcement of policy and implementation through two mechanisms of governance and leadership help to mentioned elements, so that through operations of the health system lead to achieving middle and final goals at different levels of health system. Investigated studies also did not mention this issue. Implementing this model for health have some prerequisites such as transparency, stability of policies, quality of bureaucracy, and laws and rules. For achieving stewardship goals these prerequisites should be considered. Considering these necessities as the prerequisites of stewardship are not considered in many studies. Therefore, stewardship can achieve its middle and final goals through mentioned sub-task, via an appropriate process that is mentioned in the model.

## Conclusion

It is a conceptual model that because of its comprehensiveness, covering the experience of other leading countries, using scientific resources, considering different levels of influences on health and its requirements can be used in different countries. Albeit, before implementation, mentioned & local requirements must be considered. Then, this model can be a guide for policy-makers about various sub-functions of the health stewardship at different levels of decision making, include Intra-sectoral or inter-sectoral.

## Ethical considerations

Ethical issues (Including plagiarism, informed consent, misconduct, data fabrication and/or falsification, double publication and/or submission, redundancy, etc.) have been completely observed by the authors.
